# Ultrasound-Promoted Greener Synthesis of Novel Trifurcate 3-Substituted-chroman-2,4-dione Derivatives and Their Drug-Likeness Evaluation

**DOI:** 10.3390/molecules171214146

**Published:** 2012-11-28

**Authors:** Chengyuan Liang, Hailong Jiang, Zhiguang Zhou, Dong Lei, Yu Xue, Qizheng Yao

**Affiliations:** School of Pharmacy, China Pharmaceutical University, Nanjing 210009, Jiangsu, China; Email: chengyuanliang@gmail.com (C.L.); jianghailongsj@163.com (H.J.); zhouzhiguang0728@126.com (Z.Z.); leidong0066@126.com (D.L.); xueyu0816@126.com (Y.X.)

**Keywords:** chroman-2,4-diones, multicomponent reactions (MCRs), ultrasonic irradiation, green chemistry, coumarin, edaravone

## Abstract

An efficient and convenient approach for one-pot synthesis of 3-substituted chroman-2,4-diones via a three-component reaction of aromatic aldehydes, 4-hydroxy- coumarins and diverse pyrazolone derivatives was described. The combinatorial synthesis for this methodology was achieved by applying ultrasound irradiation in the absence of activator while making use of water as green solvent. Additionally, novel chroman-2,4-dione derivatives attached to an edaravone moiety represent an exploitable source of brand new anticancer agents. In comparison with conventional methods, experimental simplicity, good functional group tolerance, excellent yields, short routine, and atom efficiency are prominent features of this sonocatalyzed procedure.

## 1. Introduction

In recent years, ultrasound-assisted organic synthesis (UAOS) utilizing environmentally benign reagents and conditions has been one of the most fascinating developments in the arena of modern synthetic chemistry owing to its green credentials [1,2,3,4,5]. The notable features of the aqueous UAOS come from its advantages such as easier manipulation, energy conservation, rate enhancement and insolubility of the final products which facilitates their isolation by simple filtration [6,7].

**Figure 1:**
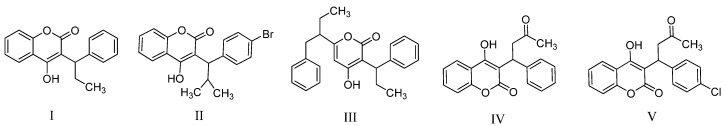
Examples of biologically active 4-hydroxycoumarin derivatives.

Coumarins, an old class of benzopyrene compounds, are widely used in the pharmaceutical industry as precursor molecules in the synthesis of various anticoagulant [8,9], anti-HIV [10,11], anti-tumor [12,13], anti-hypertension drugs [14]. Among these therapeutic properties, their cytotoxic effects on tumors were most extensively examined by a number of* in vitro* and *in vivo* experiments as well as clinical studies. Specifically, 4-hydroxycoumarin derivatives have attracted considerable attention since this class of compounds allows access to many demonstrated bioactive agents [15,16]. For example, 4-hydroxycoumarin offshoots (Figure 1) have recently been identified as novel HIV protease inhibitors (compounds **I**,**II**,**III**)[17] and anti-tumor chemicals (compounds **IV**,**V**) [18].

**Figure 2:**
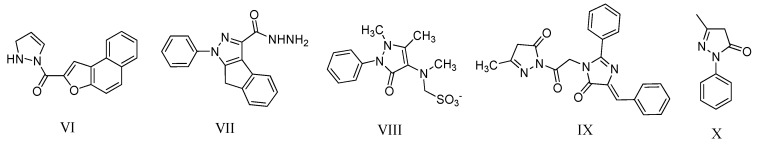
Examples of biologically active pyrazolone derivatives.

Moreover, the pyrazolone moiety (Figure 2) is an important pharmacophore which exhibits widespread pharmacological properties, such as antimicrobial (compound **VI**) [19], anticancer (compound **VII**) [20], analgesic (compound **VIII**) [21], and anti-inflammatory (compound **IX**) entities [22]. It is noteworthy that the free radical scavenger edaravone (**X**, 3-methyl-1-phenyl-2-pyrazolin-5-one, Radicut^®^) [23,24] enhances the anti-tumor effects of chemo- therapeutics in murine colon cancer by increasing apoptosis via inhibition of nuclear factor kappa B (NF-κB) [25]. Meanwhile, renal toxicity (acute cisplatin-induced renal injury) was reversed by clinically available edaravone [26,27]. Therefore, in addition to its usefulness in the treatment of strokes, edaravone is expected to play an integral role in oncotherapy.

**Scheme 1:**
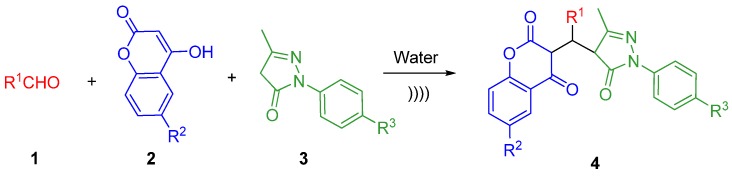
Synthesis of 3-substituted chroman-2,4-diones under ultrasonic irradiation.

From the viewpoint of rational drug design, the pharmacological properties and therapeutic applications of coumarins depend upon the pattern of substitution on the benzopyrene nucleus. Hence, novel chroman-2,4-dione (tautomer of 4-hydroxycoumarin) derivatives attached to an edaravone moiety represented an exploitable source of brand new anticancer agents, which might also help addressing side-toxicity. In the present research work, the objective was to develop new chemical entities (triplet 3-substituted chroman-2,4-diones interlinking 4-hydroxycoumarins and edaravone) as basic scaffolds, endowed with potential synergy in elevating antitumor effects as well as preventing the adverse effects and decreasing toxicity. Herein, we are delighted to afford a convenient, practical and efficient reaction for the synthesis of the target lead compounds via readily available building blocks under ultrasonic irradiation in water as shown in Scheme 1. The present research would significantly widen the versatility of the diversity-oriented aldehyde-based MCRs.

## 2. Results and Discussion

**Scheme 2:**
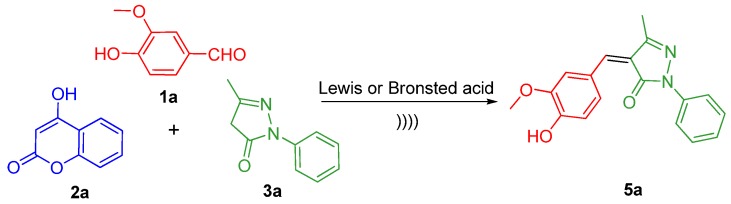
Side-reaction promoted by Lewis acids or BrØnsted acids.

To find out the suitable conditions for the reaction, our initial experiments were demonstrated by a series of experiments with the model reaction (Scheme 2) using different catalysts and solvents. In each case, the substrates were mixed together with 25 mL of solvent under ultrasonic irradiation (40 kHz) or high speed stirring with traditional heating. The screening results of the reaction are listed in Table 1.

**Table 1:** The effect of the reaction conditions for the synthesis of **4a** under ultrasound irradiation.

Entry	Solvent	Catalyst (mol%)	Temprature (°C)	With sonication ^a^		Without sonication ^b^	
				Time (h)	Yield (%) ^c^	Time (h)	Yield (%) ^c^
1	EtOH	———	70	1	55	4	23
2	Acetonitrile	———	80	1	14	4	<5
3	Toluene	———	80	0.5	—	2	—
4	Water	———	60	0.5	81	2	67
5	EtOH	ZnCl_2_(5)	70	0.5	—	2	—
6	Acetonitrile	Et_3_N(5)	80	1	Trace	4	Trace
7	Water	Morpholine(5)	60	1	36	6	15
8	Water	ZnCl_2_(5)	60	0.5	—	2	—
9	Water	AlCl_3_(5)	60	0.5	—	2	—
10	Water	Morpholine(5)	60	0.5	51	2	36
11	Water	Et_3_N(5)	60	0.5	35	2	17
12	Water	L-proline(5)	60	0.5	42	2	30

^a^ Ultrasonic frequencies of 40 kHz, while the ultrasonic power was kept at 250 W; ^b^ The mixture was kept silent under high stirring condition; ^c^ Isolated yields.

### 2.1. Effects of the Catalysts under Ultrasound Irradiation

It was observed that when classical organic bases, such as morpholine and triethylamine, were used to mediate the reaction, low yields were obtained (entries 6, 7 and 10, 11). Further study with varying Lewis acids and BrØnsted acids revealed no yields of **4a** compared with basic catalyst, while simultaneously, the byproduct **5a** was predominantly afforded by a side-reaction (Scheme 2, entries 5, 8 and 9). 

As can be seen from Table 1, the reaction rendered moderate amounts of desired product **4a** in the presence of L-proline (entry 12). When the reaction was performed in ethanol and acetonitrile in the absence of catalyst, **4a** was obtained in 55% and 14% yield, respectively (entries 1 and 2). Specifically, the formation of product **4a** was more facile and proceeded in pure water to give not only high yield, but also with high reaction rate (81% yield in 30 min, entry 4).

### 2.2. Effects of the Solvents under Ultrasound Irradiation

Polar protic solvents (ethanol and water) afforded moderate to high yields of the desired product. When the reaction was performed in acetonitrile, the desired product **4a** was only obtained in 14% yield (entry 2) and took a comparatively longer reaction time. Disappointingly, when the reaction was conducted in the non-polar solvent toluene, only the byproduct **5a** was obtained (entry 3, yield, 83%). In general, improvements in rates and yields of all trials are observed when reactions were carried out in polar protic solvents compared with polar aprotic and non-polar solvents. 

### 2.3. Comparison of Ultrasonic Irradiation and Conventional Method

As depicted in Table 1, the ultrasound-assisted technique was proven to be excellent in all cases whereas traditional heating has low efficiency, even with prolonged reaction times. Based on the results from this study, it was attractive that the ultrasound improved both the purity and yield of products. In order to verify the effect of irradiation frequency, the reaction was also performed in 30, 40, and 50 kHz. When the frequency was 40 kHz, the yield of **4a** (81%, Table 2, entry 2) was higher than that with 30 kHz irradiation within 30 min (67%, Table 2, entry 1). With an increase of irradiation frequency from 40 to 50 kHz (Table 2, entries 2, 3), the reaction yield did not change considerably (79%). The results showed that there was an optimum frequency for effective synthesis of **4a** in the frequency of 40 kHz at 60 °C. 

**Table 2:** The synthesis of **4a** under ultrasound irradiation in various frequency ^a^.

Entry	Frequency (kHz)	Temprature (°C)	Time (h)	Yield (%) ^b^
	30	60	0.5	67
2	40	60	0.5	81
3	50	60	0.5	79

^a^ *Reaction conditions*: the ultrasonic power was kept at 250 W; ^b^ Isolated yield.

### 2.4. High Efficiency and Generality of Synthesis by Ultrasound Irradiation

With the optimized conditions in hand, the versatility of this protocol was investigated by a library of sixteen 3-substituted-chroman-2,4-diones combining substituted aromatic aldehydes **1**, 4-hydroxy- coumarins **2** and pyrazolones **3**. According to Table 3, 4-hydroxycoumarins and pyrazolones carrying either electron-releasing or electron-withdrawing substituents at the *para*-position of the aromatic ring proved to be suitable substrates for this reaction. On the other hand, the aromatic aldehydes bearing electron-donating groups are more reactive in this protocol, affording the desired products with excellent yields and after shortened reaction times (Table 3, entries **3**, **5** and **7**). It is noteworthy that the hydroxyl group of vanillin remains intact after the reaction, obviously, which leaves a useful handle for further synthetic diversity.

**Table 3:** Synthesis of 3-substituted-chroman-2,4-diones(**4a**–**p**) via Scheme 1.

Entry	R_1_	R_2_	R_3_	Time (min)	Product	Isolated Yield (%)	Mp (°C)	logP ^a^
1	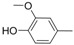	H	H	30	**4a**	81	151–153	3.34 ± 0.04
2	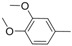	H	H	30	**4b**	89	168–170	3.60 ± 0.04
3	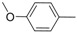	H	H	30	**4c**	83	163–165	3.73 ± 0.05
4	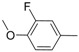	H	H	30	**4d**	77	166–168	3.89 ± 0.04
5	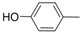	H	H	30	**4e**	82	184–186	3.47 ± 0.04
6	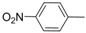	H	H	30	**4f**	71	177–179	4.17 ± 0.05
7	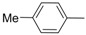	H	H	30	**4g**	78	149–151	4.34 ± 0.04
8	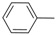	H	H	30	**4h**	83	133–135	3.86 ± 0.04
9	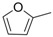	H	H	30	**4i**	65	130–132	2.45 ± 0.06
10	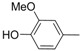	Cl	H	30	**4j**	79	195–197	3.90 ± 0.04
11	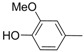	F	H	30	**4k**	58	189–191	3.51 ± 0.04
12	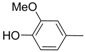	H	Me	30	**4l**	87	193–196	3.83 ± 0.05
13	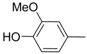	H	F	30	**4m**	73	187–189	3.50 ± 0.04
14	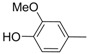	H	Cl	30	**4n**	64	185–187	4.02 ± 0.04
15	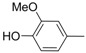	F	Me	30	**4o**	81	199–201	3.98 ± 0.06
16	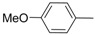	F	F	30	**4p**	83	162–164	4.06 ± 0.04

^a ^Isolated yield; ^b ^Theoretical values of logP were calculated using commercially available ACD LAB/log P release 10, product version 10.08.

The structures of isolated new products **4a-p** were determined by physical and spectroscopic data such as: ^1^H-NMR, ^13^C-NMR and ESI-HRMS analysis. Taking compound **4a **as a representative example, its ^1^H-NMR spectrum revealed the presence of two singlet signals at δ = 2.41 and δ = 3.62 ppm corresponding to the -CH_3_ group of edaravone and the -OCH_3_ group adjacent to vanillin, respectively, and another singlet signal at δ = 5.66 ppm due to the -OH of the vanillin scaffold, which underwent a facile hydrogen exchange upon addition of deuterium oxide. In the HH-COSY spectroscopy of compound **4a**, correlations between adjacent protons from three stereogenic centres were observed (4.05 ppm, 1.19 ppm and 1.17 ppm). This phenomenon suggested that the structure of compound **4a **was depicted accurately, which could be proved by the other 2D-NMR spectra.

### 2.5. the Study of Acceleration Mechanism under Irradiation of Ultrasound

**Scheme 3:**
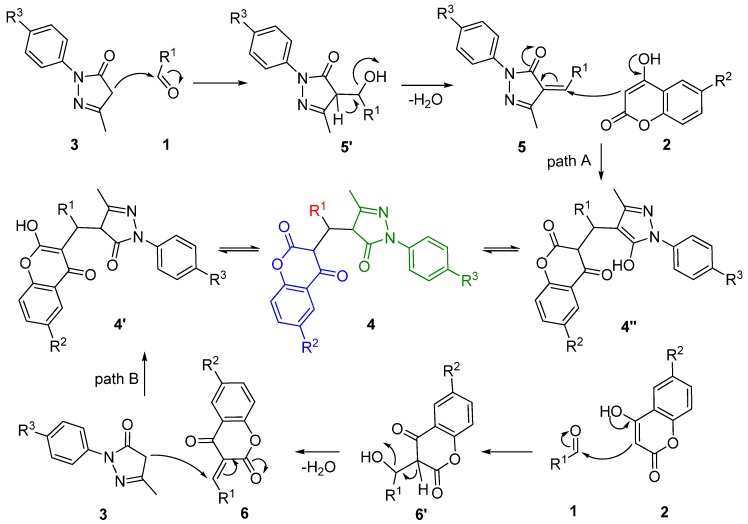
Plausible mechanism for the formation of product **4**.

A plausible mechanism is proposed and shown in Scheme 3. The process represents a typical tandem of Knoevenagel condensation and Michael addition, which might initiate via two pathways, namely path A and path B.

Once the three components are mixed, aldehyde **1** undergoes a Knoevenagel condensation with edaravone (**3**) under ultrasonic irradiation to afford the relay moiety **5 **after dehydratation, which is subsequently attacked by 4-hydroxycoumarin **2** to furnish the central target compound **4** (path A). Alternatively, intermediate **6’** is likely formed from initial condensation of **1** with **2** to afford **6**, followed by nucleophilic addition of edaravone **3** (path B).

Upon irradiation by high-intensity ultrasound, acoustic cavitation occurs and gives rise to the formation, growth, and implosive collapse of bubbles in water with consequent high local temperatures and pressures, which accelerates the mass transfer and shortens the reaction time. 

### 2.6. Drug-Likeness Evaluation of Obtained 3-substituted-chroman-2,4-Diones

Drug-likeness may be defined as a complex balance of intriguing insights, including various molecular properties and structure features, which determine whether a particular molecule has potentiality to be an orally active drug. Lipinski *et al. * [28,29] examined the drugs on the market and established the so-called “Rules of Five”, which have served as a guide in drug-like analysis and as an efficient filter in prioritizing compound libraries prior to high-throughput screening. With drug-like criteria in mind, the molecular mass of each obtained “drug-like” compound should be less than 500 Daltons. The partition coefficient logP values of the synthesized molecules were calculated (Table 3) and found to be between 2.45 and 4.34. This range is in agreement with estimated values for good lipophilicity and solubility (between 2.0 and 5.0). 

Aqueous solubility of a solid chemical can be also applied as a rule to define drug-likeness. Melting point is usually an indicator of crystal lattice energy, which has a dramatic influence on solvation free energy (aqueous solubility). The ceiling melting point of all the trifurcate 3-substituted-chroman-2,4-diones discussed above is 201 °C, in which means favorable estimated solubility. We concluded that all the key parameters (predictors of molecular transport properties across cell membranes and indicators of protein binding characteristics) have no violation of Lipinski’s Rules at all.

## 3. Experimental

### General

All reagents used in this work were obtained commercially available and were used without purification. ^1^H- and ^13^C-NMR spectra were recorded on a BRUKER AV-300 spectrometer at 300.13 and 75.47 MHz, respectively. ^1^H and ^13^C-NMR spectra were obtained from solutions of DMSO-d_6_ and are reported as parts per million (ppm) downfield from a tetramethylsilane internal standard. The following abbreviations are used; singlet (s), doublet (d), triplet (t) and multiplet (m). The mass spectrometric analyses (HRMS) were performed using a JMS-700 MStation High Resolution JEOL Mass Spectrometer with a source temperature of 230 °C, an ionization energy of 70 eV and an ionization trap current of 300 A. Melting points were measured by a differential scanning calorimeter (Shimadzu DSC-50) and were uncorrected. The standard heating rate for all compounds was 10 °C/min. Sonication was performed in Shanghai Branson-CQX ultrasonic cleaner. The flask was located at the maximum energy area in the cleaner and addition or removal of water was used to control the temperature of the water bath.

### 3.1. Typical Procedure for the Synthesis of Trifurcate 3-substituted-chroman-2,4-Dione (**4a**) in Water

A 50 mL flask was charged with vanillin (152 mg, 1 mmol), edaravone (174 mg, 1 mmol) and 4-hydroxycoumarin (162 mg, 1 mmol) in water (25 mL). The reaction conditions were adjusted separately as indicated in Table 1. After completion of the reaction (monitored by TLC)., the solvent was removed under reduced pressure, and the crude mixture was purified by silica gel column chromatography (*n*-hexane/ethyl acetate, 20:80) to offer pure product **4a**.

### 3.2. Ultrasound-promoted Typical Procedure for Synthesis of 3-substituted-chroman-2,4-dione (**4a**)

A 50 mL flask was charged with vanillin (152 mg, 1 mmol), edaravone (174 mg, mmol) and 4-hydroxycoumarin (162 mg, 1mmol) in water (25 mL). The reaction flask was placed in the ultrasonic bath, where the surface of reactants is slightly lower than the level of the water, and irradiated separately under 30, 40, and 50 kHz at 50 °C (bath temperature, the temperature inside the reactor was also 50 °C) for the period of time as indicated in Table 1, Table 2, Table 3. The reaction temperature was controlled by addition or removal of water from ultrasonic bath. After completion of the reaction (monitored by TLC), the crude mixture was purified by silica gel column chromatography to afford desired product **4a** after solvent evaporation.

### 3.3. Spectral Data for New Derivatives of Trifurcate 3-substituted-chroman-2,4-diones **4a–p**

*3-((4-Hydroxy-3-methoxyphenyl)(3-methyl-5-oxo-1-phenyl-4,5-dihydro-1H-pyrazol-4-yl)methyl)chroman-2,4-dione* (**4a**). Pale yellow crystals;^ 1^H-NMR (DMSO-d_6_) δ 7.84 (1H, d, *J *= 7.28 Hz), 7.72 (2H, d, *J *= 7.28 Hz), 7.59–7.50 (3H, m), 7.38–7.31 (3H, m), 6.73–6.64 (3H, m), 5.65 (1H, s), 4.05 (1H, dd, *J* = 4.30 Hz, *J* = 5.00 Hz), 3.62 (3H,s), 2.41 (3H,s), 1.19 (1H, d, *J* = 5.00 Hz), 1.17 (1H, d, *J* = 4.30 Hz); ^13^C-NMR (DMSO-d_6_) δ 164.7, 163.8, 162.7, 152.4, 147.7, 145.5, 135.7, 132.3, 130.7, 129.7, 127.0, 124.3, 121.2, 119.9, 118.5, 116.3, 115.7, 112.2, 107.5, 106.4, 60.2, 56.2, 33.7, 21.1, 14.5; HRMS (ESI) for (M+H)^+^: calcd 471.1 556, found 471.1559.

*3-((3,4-Dimethoxyphenyl)(3-methyl-5-oxo-1-phenyl-4,5-dihydro-1H-pyrazol-4-yl)methyl)chroman-2,4-dione *(**4b**). Pale yellow crystals;^ 1^H-NMR (DMSO-d_6_) δ: 7.84 (2H, d, *J* = 7.10 Hz), 7.75 (1H, d, *J* = 7.10 Hz), 7.57 (1H, m), 7.49–7.31 (4H, m), 7.13–6.81 (4H, m), 4.20 (1H, dd, *J* = 5.10 Hz, *J* = 4.40 Hz), 3.74 (6H, s), 2.42 (3H, s), 1.20 (1H, d, *J* = 5.10 Hz), 1.16(1H, d, *J* = 4.40 Hz); ^13^C-NMR (DMSO-d_6_) δ: 169.0, 162.5, 161.2, 155.6, 152.5, 147.5, 145.0, 136.7, 133.5, 129.9, 128.0, 127.6, 124.6, 124.5, 119.4, 117.3, 112.1, 109.5, 58.6, 56.1, 34.3, 22.0, 15.7; HRMS (ESI) for (M+H)^+^: calcd 485.1713, found 485.1719.

*3-((4-Methoxyphenyl)(3-methyl-5-oxo-1-phenyl-4,5-dihydro-1H-pyrazol-4-yl)methyl)chroman-2,4-dione *(**4c**). Colorless crystals; ^1^H-NMR (DMSO-d_6_) δ: 7.94 (2H, d, *J* = 7.10 Hz ), 7.78 (2H, d, *J* = 7.10 Hz), 7.60–7.51 (4H, m), 7.48–7.34 (4H, m), 7.19 (1H, m), 4.15 (1H, dd, *J* = 4.30 Hz, *J* = 3.81 Hz), 3.83 (3H, s), 2.44(3H, s), 1.20 (1H, d, *J* = 4.30 Hz), 1.17 (1H, d, *J* = 3.81 Hz); ^13^C-NMR (DMSO-d_6_) δ: 173.9, 169.0, 162.5, 161.2, 157.8, 155.6, 152.5, 140.7, 138.9, 133.5, 128.9, 128.0, 125.4, 124.6, 124.5, 119.4, 117.3, 115.2, 59.2, 56.1, 39.2, 21.7, 14.9; HRMS (ESI) for (M+H)^+^: calcd 455.1607, found 455.1611.

*3-((3-Fluoro-4-methoxyphenyl)(3-methyl-5-oxo-1-phenyl-4,5-dihydro-1H-pyrazol-4-yl)methyl)chroman-2,4-dione *(**4d**). Colorless crystals; ^1^H-NMR (DMSO-d_6_) δ: 7.88(1H, d, *J* = 6.82 Hz), 7.74 (2H, d, *J* = 6.82 Hz), 7.58–7.34 (5H, m), 7.19–6.73 (4H, m), 4.17 (1H, dd, *J* = 3.95 Hz, *J* = 2.50 Hz), 3.83 (3H, s), 2.43 (3H, s) 1.20 (1H, d, *J* = 2.50 Hz ), 1.18 (1H, d, *J* = 3.95 Hz); ^13^C-NMR (DMSO-d_6_) δ: 175.1, 169.0, 162.9, 160.3, 155.6, 152.5, 151.8, 144.9, 142.3, 138.4, 132.5, 129.4, 127.6, 126.0, 125.4, 124.7, 124.2, 119.4, 117.3, 115.1, 108.1, 58.6, 55.8, 41.2, 21.7, 14.1; HRMS (ESI) for (M+H)^+^: calcd 473.1513, found 473.1518.

*3-((4-Hydroxyphenyl)(3-methyl-5-oxo-1-phenyl-4,5-dihydro-1H-pyrazol-4-yl)methyl)chroman-2,4-dione* (**4e**). Light red crystals; ^1^H-NMR (DMSO-d_6_) δ: 7.89 (1H, d, *J* = 7.15 Hz), 7.78 (2H, d, *J* = 7.15 Hz), 7.60–7.36 (5H, m), 7.29–7.03 (3H, m), 6.67 (2H, m), 5.85 (1H, s), 4.12 (1H, dd, *J* = 3.80 Hz, *J* = 2.50 Hz), 2.44 (3H, s) 1.19 (1H, d, *J* = 2.50 Hz), 1.16 (1H, d, *J* = 3.80 Hz); ^13^C-NMR (DMSO-d_6_) δ: 172.9, 169.0, 166.6, 162.8, 155.7, 155.6, 153.5, 140.1, 138.9, 135.6, 133.5, 128.9, 127.5, 124.6, 117.3, 115.6, 110.7, 108.6, 58.6, 43.2, 21.7, 15.7; HRMS (ESI) for (M+H)^+^: calcd 441.1450, found 441.1459.

*3-((3-Methyl-5-oxo-1-phenyl-4,5-dihydro-1H-pyrazol-4-yl)(4-nitrophenyl)methyl)chroman-2,4-dione* (**4f**). Brown crystals; ^1^H-NMR (DMSO-d_6_) δ: 8.28–7.96 (4H, m), 7.78–7.60 (2H, m), 7.55–7.03 (7H, m), 4.22 (1H, dd, *J* = 4.80 Hz, *J* = 3.70 Hz), 2.43 (3H, s), 1.19 (1H, d, *J* = 3.70 Hz), 1.17 (1H, d, *J* = 4.80 Hz); ^13^C-NMR (DMSO-d_6_) δ: 178.9, 169.0, 166.3, 161.8, 155.6, 152.5, 145.1, 143.2, 138.9, 133.5, 128.9, 127.6, 127.5, 125.4, 124.6, 123.6, 117.3, 109.2, 58.6 , 46.2, 21.7, 19.9; HRMS (ESI) for (M+H)^+^: calcd 470.1352, found 470.1356.

*3-((3-Methyl-5-oxo-1-phenyl-4,5-dihydro-1H-pyrazol-4-yl)(p-tolyl)methyl)chroman-2,4-dione *(**4g**). Colorless crystals; ^1^H-NMR (DMSO-d_6_) δ: 7.98–7.70 (3H, m), 7.60–7.30 (7H, m), 7.09–6.80 (3H, m), 4.13 (1H, dd, *J* = 4.40 Hz, *J* = 3.51 Hz), 2.43 (3H, s), 1.94 (3H, s), 1.19 (1H, d, *J* = 3.51 Hz), 1.17 (1H, d, *J* = 4.40 Hz); ^13^C-NMR (DMSO-d_6_) δ: 170.9, 168.0, 163.5, 160.2, 158.3, 157.8, 155.6, 152.5, 140.7, 138.9, 133.5, 128.9, 128.0, 125.4, 124.6, 124.5, 119.4, 117.3, 115.2, 58.6, 56.1, 39.2, 20.8, 14.9; HRMS (ESI) for (M+H)^+^: calcd 439.1658, found 439.1655.

*3-((3-Methyl-5-oxo-1-phenyl-4,5-dihydro-1H-pyrazol-4-yl)(phenyl)methyl)chroman-2,4-dione *(**4h**). Colorless crystals; ^1^H-NMR (DMSO-d_6_) δ: 7.88 (2H, d, *J* = 6.10 Hz), 7.73 (1H, d, *J* = 6.10 Hz), 7.59 (1H, t, *J* = 6.10 Hz), 7.48–7.30 (8H, m), 7.27–7.19 (2H, m), 4.08 (1H, dd, *J* = 4.60 Hz, *J* = 3.91 Hz), 2.40 (3H, s) 1.20 (1H, d, *J* = 3.91 Hz), 1.17 (1H, d, *J* = 4.60 Hz); ^13^C-NMR (DMSO-d_6_) δ: 178.9, 172.9, 169.0, 166.5, 155.6, 152.5, 148.2, 138.9, 135.6, 133.2, 129.4, 128.1, 125.4, 124.6, 122.2, 117.3, 114.0, 106.5, 56.8, 46.2, 20.7, 14.3; HRMS (ESI) for (M+H)^+^: calcd 424.1423, found 424.1427.

*3-(Furan-2-yl(3-methyl-5-oxo-1-phenyl-4,5-dihydro-1H-pyrazol-4-yl)methyl)chroman-2,4-dione *(**4i**). Colorless crystals; ^1^H-NMR (DMSO-d_6_) δ: 7.85 (2H, d, *J* = 6.96 Hz ), 7.73 (1H, d, *J* = 6.96 Hz), 7.58 (1H, m), 7.48–7.03 (6H, m), 6.40 (1H, t, *J* = 6.30 Hz), 6.08 (1H, t, *J* = 6.30 Hz), 4.40 (1H, dd, *J* = 3.90 Hz, *J* = 2.80 Hz), 2.42 (3H, s), 1.20 (1H, d, *J* = 2.80 Hz), 1.16 (1H, d, *J* = 3.90 Hz); ^13^C-NMR (DMSO-d_6_) δ: 179.1, 174.8, 169.3, 167.5,161.3, 155.6, 152.5, 148.2, 138.9, 135.6, 133.2, 129.4, 128.0, 127.6, 125.4, 124.6, 117.3, 110.0, 107.3, 104.7, 56.8, 44.4, 22.9, 19.3; HRMS (ESI) for (M+H)^+^: calcd 415.1294, found 415.1293.

*6-Chloro-3-((4-hydroxy-3-methoxyphenyl)(3-methyl-5-oxo-1-phenyl-4,5-dihydro-1H-pyrazol-4-yl)methyl)chroman-2,4-dione *(**4j**). Pale yellow crystals;^ 1^H-NMR (DMSO-d_6_) δ: 8.05–7.66 (4H, m), 7.43–7.05 (4H, m), 6.83–6.66 (3H, m), 5.45(1H, s), 4.05 (1H, dd, *J* = 4.40 Hz, *J* = 3.60 Hz), 3.62 (3H, s), 2.44 (3H, s), 1.21 (1H, d, *J* = 3.60 Hz), 1.17(1H, d, *J* = 4.40 Hz); ^13^C-NMR (DMSO-d_6_) δ:174.7, 168.8, 165.3, 156.5, 149.7, 145.6, 135.9, 131.3, 130.7, 128.7, 127.3, 125.1, 121.7, 119.5, 118.3, 116.2, 115.1, 112.8, 107.2, 106.2, 60.9, 56.2, 34.7, 22.1, 15.3; HRMS (ESI) for (M+H)^+^: calcd 505.9264, found 505.9267.

*6-Fluoro-3-((4-hydroxy-3-methoxyphenyl)(3-methyl-5-oxo-1-phenyl-4,5-dihydro-1H-pyrazol-4-yl)methyl)chroman-2,4-dione *(**4k**). Pale yellow crystals;^ 1^H-NMR (DMSO-d_6_) δ: 7.95–7.75 (3H, m), 7.44–7.07 (5H, m), 6.84–6.68 (3H, m), 5.35 (1H, s), 4.12 (1H, dd, *J* = 4.10 Hz, *J* = 3.30 Hz), 3.65 (3H, s), 2.43 (3H, s), 1.20 (1H, d, *J* = 4.10 Hz), 1.18 (1H, d, *J* = 3.30 Hz); ^13^C-NMR (DMSO-d_6_) δ:178.7, 167.7, 166.2, 158.3, 150.1, 146.2, 136.8, 133.1, 131.4, 129.2, 128.1, 126.8, 126.0, 118.8, 117.1, 116.1, 115.7, 111.9, 108.1, 107.4, 59.4, 55.5, 35.6, 23.1, 14.8; HRMS (ESI) for (M+H)^+^: calcd 489.1462, found 489.1466.

*3-((4-Hydroxy-3-methoxyphenyl)(3-methyl-5-oxo-1-p-tolyl-4,5-dihydro-1H-pyrazol-4-yl)methyl)chroman-2,4-dione* (**4l**). Pale yellow crystals;^ 1^H-NMR (DMSO-d_6_) δ: 7.79–7.64 (2H, m), 7.59–7.50 (3H, m), 7.38–7.31 (3H, m), 6.73–6.64 (3H, m), 5.65 (1H, s), 4.05 (1H, dd, *J* = 4.30 Hz, *J* = 3.81 Hz), 3.62 (3H, s), 2.41 (3H, s), 2.33 (3H, s), 1.20 (1H, d, *J* = 3.81 Hz), 1.18 (1H, d, *J* = 4.30 Hz); ^13^C-NMR (DMSO-d_6_) δ:167.3, 164.2, 163.4, 152.4, 147.7, 144.1, 135.7, 132.3, 131.3, 129.7, 126.9, 123.9, 121.0, 118.8, 116.3, 115.7, 114.5, 113.2, 107.4, 105.8, 61.2, 56.2, 33.7, 23.4, 21.3, 14.6; HRMS (ESI) for (M+H)^+^: calcd 485.1713, found 485.1719.

*3-((1-(4-Fluorophenyl)-3-methyl-5-oxo-4,5-dihydro-1H-pyrazol-4-yl)(4-hydroxy-3-methoxyphenyl)- methyl)chroman-2,4-dione* (**4m**). Pale yellow crystals;^ 1^H-NMR (DMSO-d_6_) δ: 7.78–7.66 (3H, m), 7.60 (1H, m), 7.48–7.05 (4H, m), 6.78–6.69 (3H, m), 5.45 (1H, s), 4.15 (1H, dd, *J* = 4.50Hz, *J* = 3.90 Hz), 3.62 (3H, s), 2.41 (3H, s), 1.19 (1H, d, *J* = 3.90 Hz), 1.17(1H, d, *J* = 4.50 Hz); ^13^C-NMR (DMSO-d_6_) δ:167.7, 164.8, 162.7, 152.4, 147.7, 145.5, 135.7, 132.3, 131.7, 129.7, 128.0, 124.3, 121.2, 120.5, 118.5, 117.3, 115.7, 112.2, 108.5, 106.4, 60.2, 58.2, 33.7, 21.1, 14.5; HRMS (ESI) for (M+H)^+^: calcd 489.1462, found 489.1467.

*1-(4-Chlorophenyl)-4-((4-hydroxy-3-methoxyphenyl)(4-oxochroman-3-yl)methyl)-3-methyl-1H-pyrazol-5(4H)-one* (**4n**). Pale brown crystals;^ 1^H-NMR (DMSO-d_6_) δ: 7.79–7.60 (4H, m), 7.46–7.04 (4H, m), 6.80–6.71 (3H, m), 5.43 (1H, s), 4.12 (1H, dd, *J* = 4.30Hz, *J* = 3.60 Hz), 3.69 (3H, s), 2.43 (3H, s), 1.20 (1H, d, *J* = 3.60 Hz), 1.18 (1H, d, *J* = 4.30 Hz); ^13^C-NMR (DMSO-d_6_) δ:169.3, 166.2, 163.7, 151.4, 148.7, 145.3, 135.4, 132.7, 131.2, 129.7, 128.2, 124.4, 121.7, 120.1, 118.4, 117.2,115.6, 112.1, 108.3, 106.2, 60.1, 58.1, 33.5, 21.0, 15.4; HRMS (ESI) for (M+H)^+^: calcd 491.1374, found 491.1378.

*6-Fluoro-3-((4-hydroxy-3-methoxyphenyl)(3-methyl-5-oxo-1-p-tolyl-4,5-dihydro-1H-pyrazol-4-yl)- methyl)chroman-2,4-dione* (**4o**). Pale yellow crystals;^ 1^H-NMR (DMSO-d_6_) δ: 7.79–7.64 (2H, m), 7.69–7.49 (3H, m), 7.39–7.30 (2H, m), 6.76–6.65 (3H, m), 5.62 (1H, s), 4.09 (1H, dd, *J* = 5.00Hz, *J* = 4.10 Hz), 3.68 (3H, s), 2.43 (3H, s), 2.31 (3H, s), 1.21 (1H, d, *J* = 4.10 Hz), 1.17 (1H, d, *J* = 5.00 Hz); ^13^C-NMR (DMSO-d_6_) δ:168.7, 165.1, 164.3, 151.2, 149.3, 145.9, 136.2, 135.7, 132.3, 130.7, 128.4, 126.1, 120.2, 118.3, 116.5, 115.2, 114.0, 113.1, 107.2, 105.0, 61.1, 57.6, 36.0, 22.9, 21.2, 14.5; HRMS (ESI) for (M+H)^+^: calcd 503.1618, found 503.1615.

*6-Fluoro-3-((1-(4-fluorophenyl)-3-methyl-5-oxo-4,5-dihydro-1H-pyrazol-4-yl)(4-methoxyphenyl)- methyl)-chroman-2,4-dione *(**4p**). Colorless crystals; ^1^H-NMR (DMSO-d_6_) δ: 7.85 (2H, d, *J* = 7.24 Hz ), 7.52 (1H, s), 7.40–7.22 (4H, m), 6.91 (4H, d, *J* = 7.24 Hz), 4.08 (1H, dd, *J* = 4.30 Hz, *J* = 3.81 Hz), 3.87 (3H, s), 2.48 (3H, s), 1.19 (1H, d, *J* = 3.81 Hz), 1.16 (1H, d, *J* = 4.30 Hz); ^13^C-NMR (DMSO-d_6_) δ: 173.9, 172.9, 169.0, 162.9, 159.6, 157.8, 155.6, 148.1, 147.2, 140.7, 138.9, 133.5, 128.9, 128.0, 125.1, 123.2, 120.2, 115.7, 114.0, 108.9, 56.3, 52.8, 39.2, 22.0, 14.9; HRMS (ESI) for (M+H)^+^: calcd 491.1419, found 491.1421.

*4-(4-Hydroxy-3-methoxybenzylidene)-3-methyl-1-phenyl-1H-pyrazol-5(4H)-one* (**5a**). Colorless crystals; Mp, 131–133°C ; ^1^H-NMR (DMSO-d_6_) δ: 9.17 (1H, s), 7.95 (1H, d, *J* = 6.80 Hz ), 7.82 (1H, d, *J* = 7.10 Hz), 7.52 (1H, t, *J* = 7.10 Hz), 7.43–7.33 (3H, m), 7.26 (1H, d, *J* = 6.80 Hz), 7.17 (1H, s), 7.01 (1H, s), 4.03 (3H, s), 2.32 (3H, s); ^13^C-NMR (DMSO-d_6_) δ: 190.8, 162.4, 151.0, 147.4, 146.4, 138.5, 131.1, 128.7, 127.4, 126.5, 124.8, 124.5, 119.4, 115.3, 114.4, 108.8, 77.4, 77.0, 76.6, 56.5, 56.1, 29.6, 13.3; HRMS (ESI) for (M+H)^+^: calcd 309.1239, found 309.1245.

## 4. Conclusions

In summary, an expeditious, greener and atom economic methodology for the synthesis of novel 3-substituted-chroman-2,4-diones has been reported. Compared with traditional synthetic methods, the application of water as a promising solvent without any harmful catalysts has received considerable attention owing to its green credentials. The aqueous ultrasound promoted synthetic approach revealed in our research was in line with the trend of developing green chemistry using environmentally benign reagents and energy economical conditions. Further biological evaluation of these modified coumarin candidates with promising improved therapeutic activity and reduced toxicity is ongoing in our laboratory. The present work could find extensive applications in view of the power of the diversity-oriented multicomponent reactions as a valid and green alternative which would widen significantly the versatility and scope of the aldehyde-based MCRs.

## Supplementary Materials

Supplementary materials can be accessed at: http://www.mdpi.com/1420-3049/17/12/14146/s1.

## References

[1] Blaskovicova M., Gaplovsky A., Blasko J. (2007). Synthesis and Photochemistry of 1-Iodocyclohexene: Influence of Ultrasound on Ionic *vs. *Radical Behaviour. Molecules.

[2] Doan N., Le T., Nguyen H., Hansen P., Duus F. (2007). Ultrasound Assisted Synthesis of 5,9-Dimethylpentadecane and 5,9-Dimethylhexadecane-The Sex Pheromones of Leucoptera coffeella. Molecules.

[3] Xiao L., Liu C.-J., Li Y.-P. (2009). Ultrasound Promoted Synthesis of Bis (substituted pyrazol-4-ylcarbonyl)-Substituted Thioureas. Molecules.

[4] Liu C.-J., Wang J.-D. (2010). Ultrasound-Assisted Synthesis of Novel 4-(2-Phenyl-1,2,3-Triazol-4-yl)-3,4-Dihydropyrimidin-2(1H)-(Thio)ones Catalyzed by Sm(ClO4)3. Molecules.

[5] Wang J., Bai X., Xu C., Wang Y., Lin W., Zou Y., Shi D. (2012). Ultrasound-Promoted One-Pot, Three-Component Synthesis of Spiro[indoline-3,1'-pyrazolo[1,2-b]phthalazine] Derivatives. Molecules.

[6] Gao D.-M., Ma W.-L., Li T.-R., Huang L.-Z., Du Z.-T. (2012). An Improved Synthesis of 1,2-Diarylethanols under Conventional Heating and Ultrasound Irradiation. Molecules.

[7] Gomha S.M., Khalil K.D. (2012). A Convenient Ultrasound-Promoted Synthesis of Some New Thiazole Derivatives Bearing a Coumarin Nucleus and Their Cytotoxic Activity. Molecules.

[8] Aggeler P.M., O'Reilly R.A., Leong L., Kowitz P.E. (1967). Potentiation of anticoagulant effect of warfarin by phenylbutazone. N. Engl. J. Med..

[9] O'Reilly R.A., Aggeler P.M. (1968). Studies on coumarin anticoagulant drugs: Initiation of warfarin therapy without a loading dose. Circulation.

[10] Huang L., Yuan X., Yu D., Lee K., Ho Chen C. (2005). Mechanism of action and resistant profile of anti-HIV-1 coumarin derivatives. Virology.

[11] Spino C., Dodier M., Sotheeswaran S. (1998). Anti-HIV coumarins from Calophyllum seed oil. Bioorg. Med. Chem. Lett..

[12] Baba M., Jin Y., Mizuno A., Suzuki H., Okada Y., Takasuka N., Tokuda H., Nishino H., Okuyama T. (2002). Studies on Cancer Chemoprevention by Traditional Folk Medicines XXIV.-Inhibitory Effect of a Coumarin Derivative, 7-Isopentenyloxycoumarin, against Tumor-Promotion. Biol. Pharm. Bull..

[13] Murakami A., Kuki W., Takahashi Y., Yonei H., Nakamura Y., Ohto Y., Ohigashi H., Koshimizu K. (1997). Auraptene, A Citrus Coumarin, Inhibits 12-o-Tetradecanoylphorbol-13-acetate-induced Tumor Promotion in ICR Mouse Skin, Possibly through Suppression of Superoxide Generation in Leukocytes. Cancer Sci..

[14] Kelly R.A., Gelfand J.A., Pincus S.H. (1981). Cutaneous necrosis caused by systemicallyadministered heparin. JAMA.

[15] Stanchev S., Momekov G., Jensen F., Manolov I. (2008). Synthesis, computational study and cytotoxic activity of new 4-hydroxycoumarin derivatives. Eur. J. Med. Chem..

[16] Chohan Z.H., Shaikh A.U., Rauf A., Supuran C.T. (2006). Antibacterial, antifungal and cytotoxic properties of novel N-substituted sulfonamides from 4-hydroxycoumarin. J. Enzym. Inhib. Med. Chem..

[17] Thaisrivongs S., Watenpaugh K.D., Howe W.J., Tomich P.K., Dolak L.A., Chong K.T., Tomich C.S.C., Tomasselli A.G., Turner S.R. (1995). Structure-based design of novel HIV protease inhibitors: carboxamide-containing 4-hydroxycoumarins and 4-hydroxy-2-pyrones as potent nonpeptidic inhibitors. J. Med. Chem..

[18] Kostova I. (2005). Synthetic and natural coumarins as cytotoxic agents. Curr. Med. Chem..

[19] Dias L.R.S., Salvador R.R.S. (2012). Pyrazole Carbohydrazide Derivatives of Pharmaceutical Interest. Pharmaceuticals.

[20] Wang X.H., Wang X.K., Liang Y.J., Shi Z., Zhang J.Y., Chen L.M., Fu L.W. (2010). A cellbased screen for anticancer activity of 13 pyrazolone derivatives. Chin. J. Cancer.

[21] Chandrasekharan N., Dai H., Roos K., Evanson N.K., Tomsik J., Elton T.S., Simmons D.L. (2002). COX-3, A cyclooxygenase-1 variant inhibited by acetaminophen and other analgesic/antipyretic drugs: Cloning, Structure, And expression. Proc. Nat. Acad. Sci. USA.

[22] Laporte J., Carne X., Vidal X., Moreno V., Juan J. (1991). Upper gastrointestinal bleeding in relation to previous use of analgesics and non-steroidal anti-inflammatory drugs. Lancet.

[23] Shichinohe H., Kuroda S., Yasuda H., Ishikawa T., Iwai M., Horiuchi M., Iwasaki Y. (2004). Neuroprotective effects of the free radical scavenger Edaravone (MCI-186) in mice permanent focal brain ischemia. Brain Res..

[24] Toyoda K., Fujii K., Kamouchi M., Nakane H., Arihiro S., Okada Y., Ibayashi S., Iida M. (2004). Free radical scavenger, edaravone, in stroke with internal carotid artery occlusion. J. Neurol. Sci..

[25] Kokura S., Yoshida N., Sakamoto N., Ishikawa T., Takagi T., Higashihara H., Nakabe N., Handa O., Naito Y., Yoshikawa T. (2005). The radical scavenger edaravone enhances the anti-tumor effects of CPT-11 in murine colon cancer by increasing apoptosis via inhibition of NF-κB. Cancer Lett..

[26] Sueishi K., Mishima K., Makino K., Itoh Y., Tsuruya K., Hirakata H., Oishi R. (2002). Protection by a radical scavenger edaravone against cisplatin-induced nephrotoxicity in rats. Eur. J. Pharmacol..

[27] Iguchi T., Nishikawa M., Chang B.J., Muroya O., sato E.F., Nakatani T., Inoue M. (2004). Edaravone inhibits acute renal injury and cyst formation in cisplatin-treated rat kidney. Free Radic. Res..

[28] Hopkins A.L., Groom C.R. (2002). The druggable genome. Nat. Rev. Drug Discov..

[29] Lipinski C.A. (2000). Drug-like properties and the causes of poor solubility and poor permeability. J. Pharmacol. Toxicol. Method..

