# Supplementary material for: Ultrasound-Promoted Greener Synthesis of Novel Trifurcate 3-Substituted-chroman-2,4-dione Derivatives and Their Drug-Likeness Evaluation
Source: Molecules. 2012 Nov 28;17(12):14146–58. doi: 10.3390/molecules171214146 (PMC6268048; doi:10.3390/molecules171214146)
Supplement: Supplementary file 1 [file molecules-17-14146-s001.pdf]

Supporting Information

Spectra of Representative 4a

Figure S1. 1H-NMR spectrum of compound **4a**.

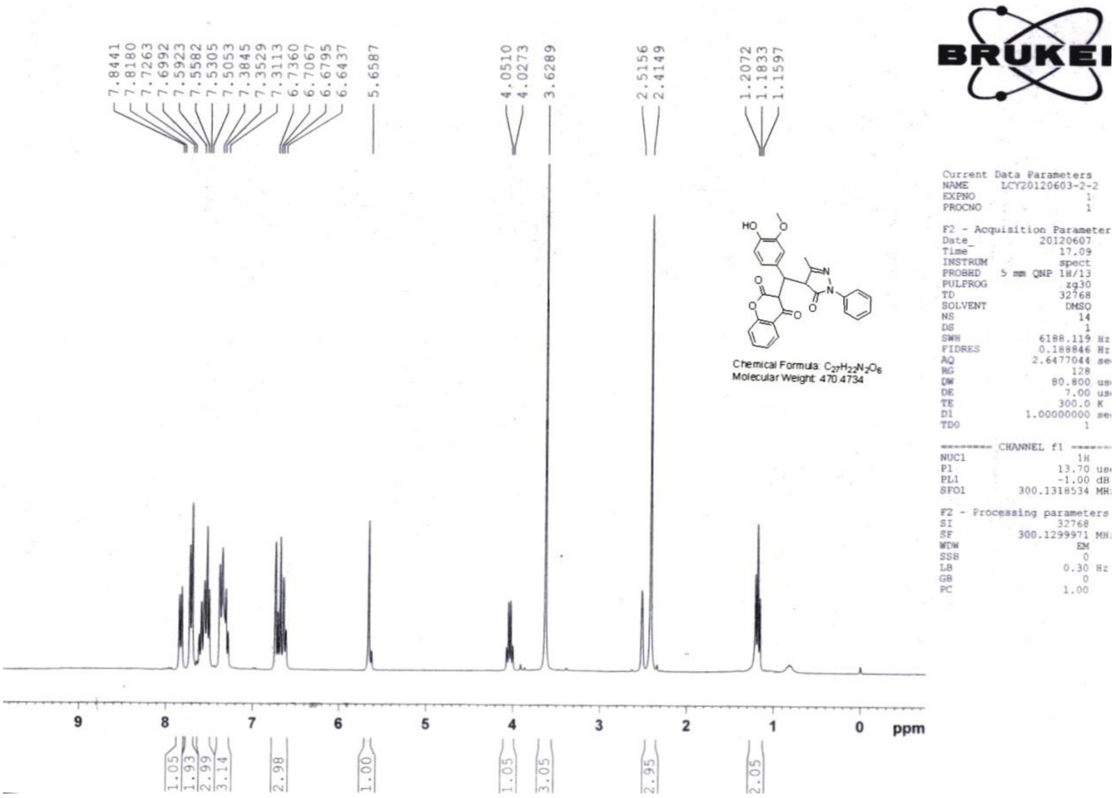

Figure S2. ^{13}C-NMR spectrum of compound **4a**.

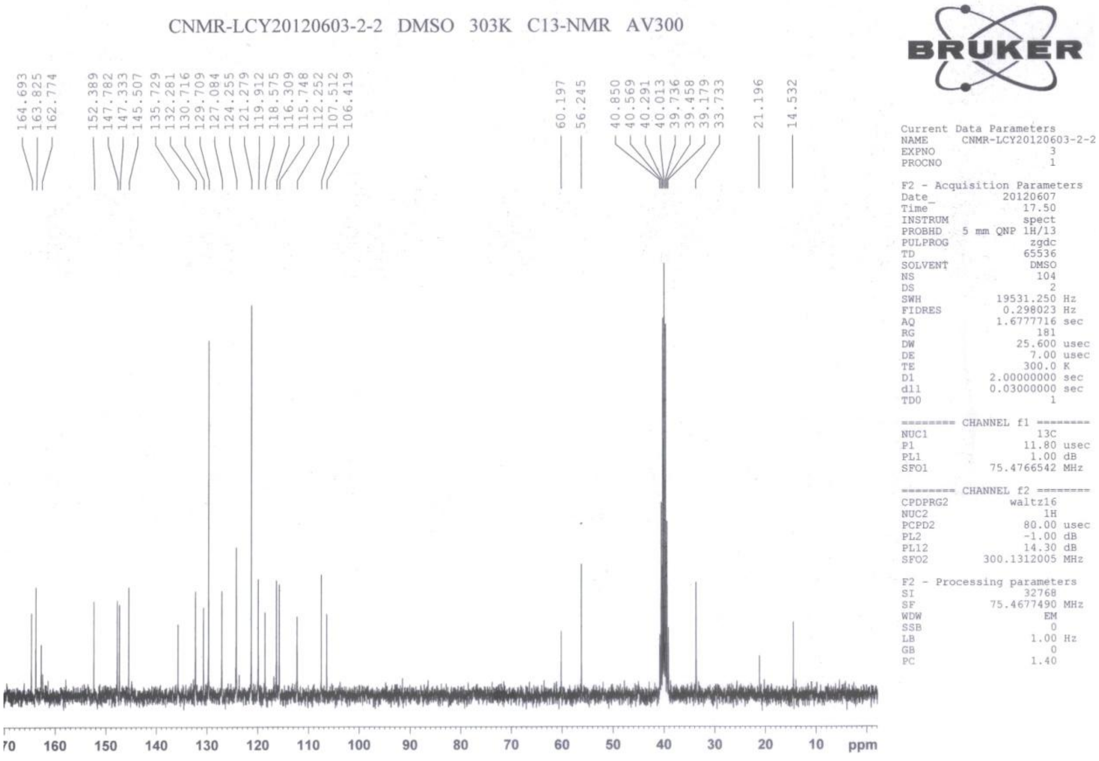

Figure S3. HSQC spectrum of compound **4a**.

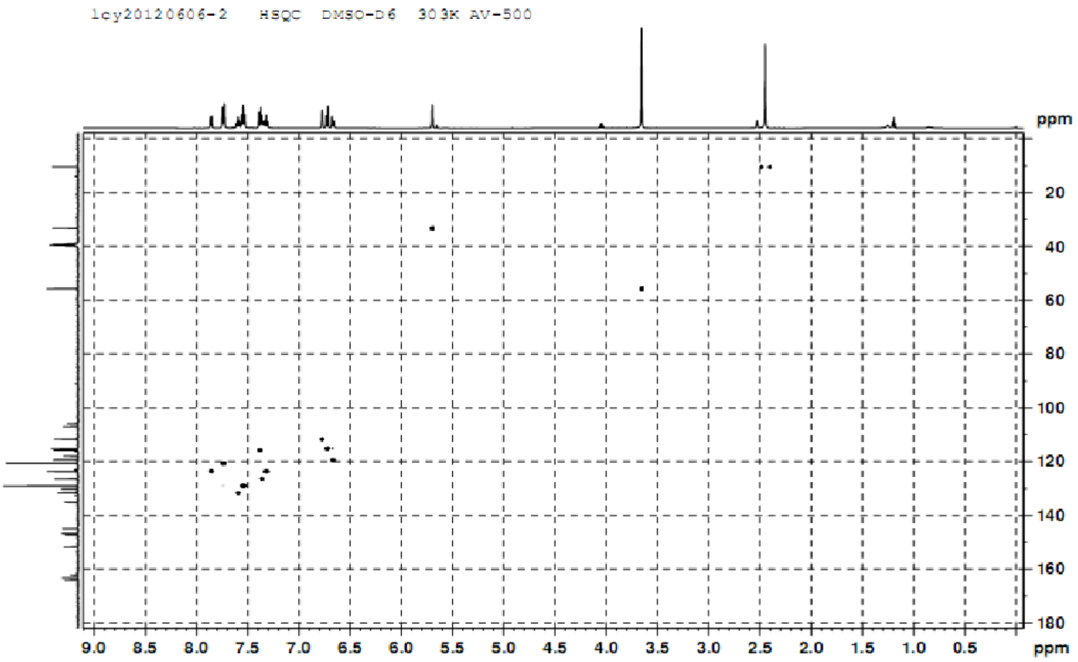

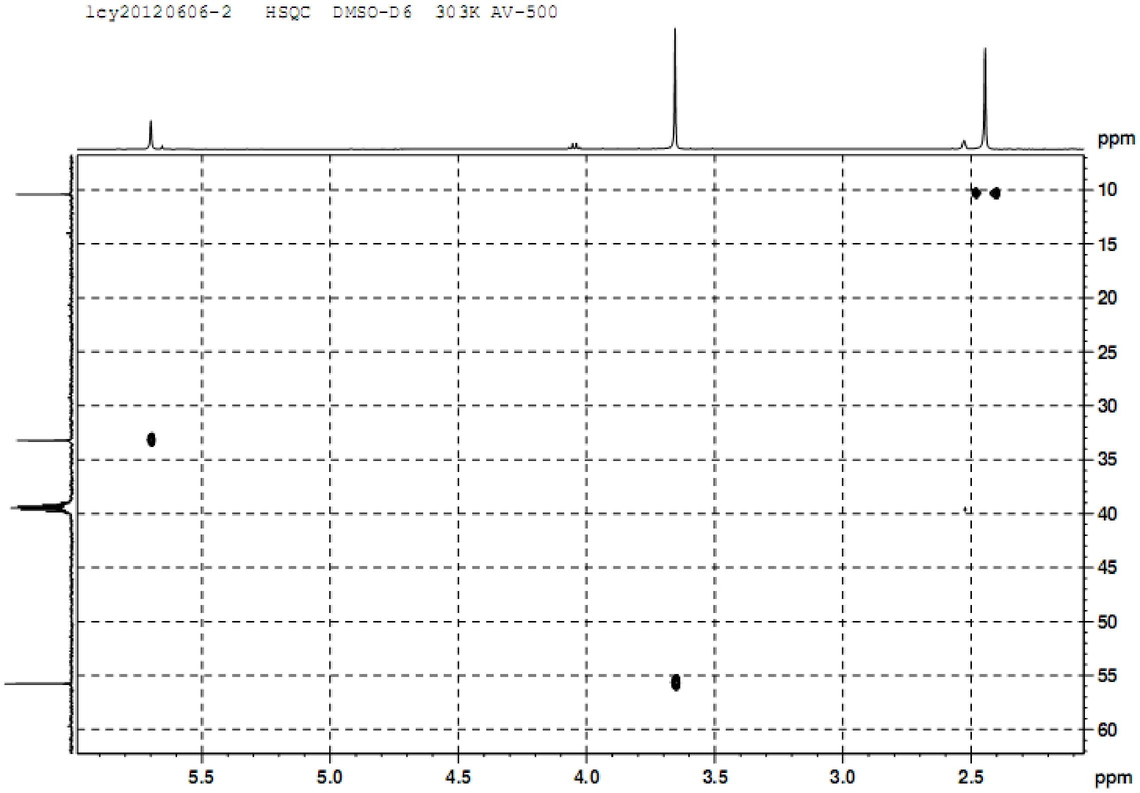

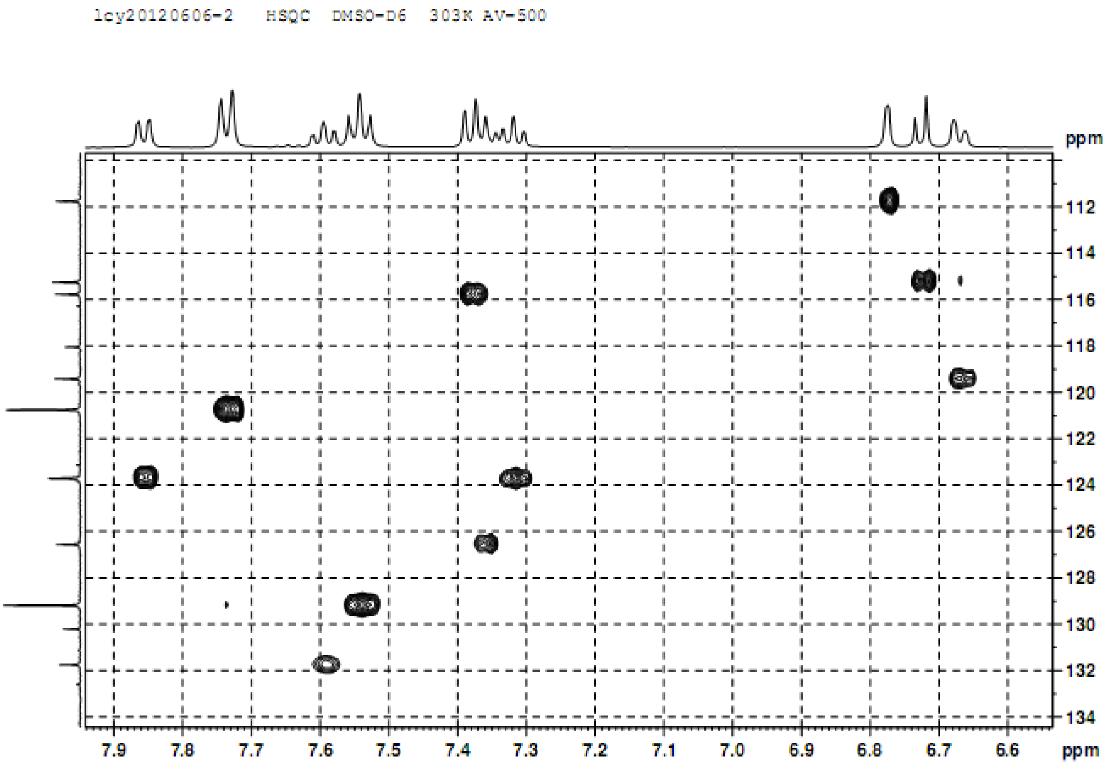

Figure S4. ROESY spectrum of compound **4a**.

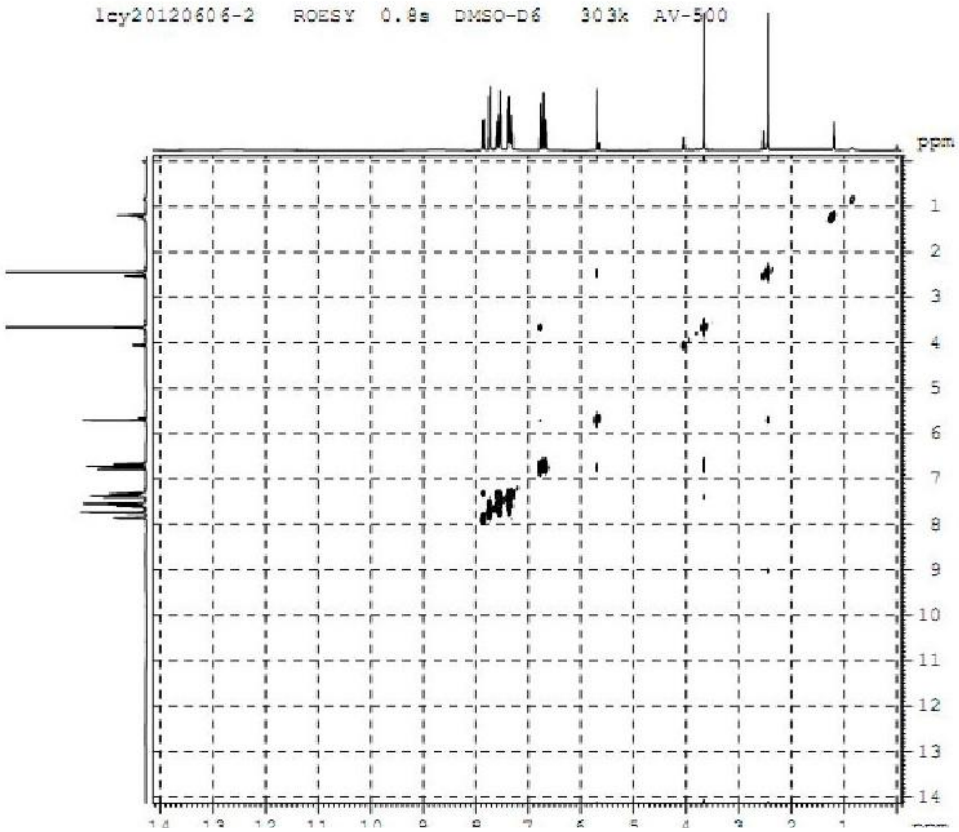

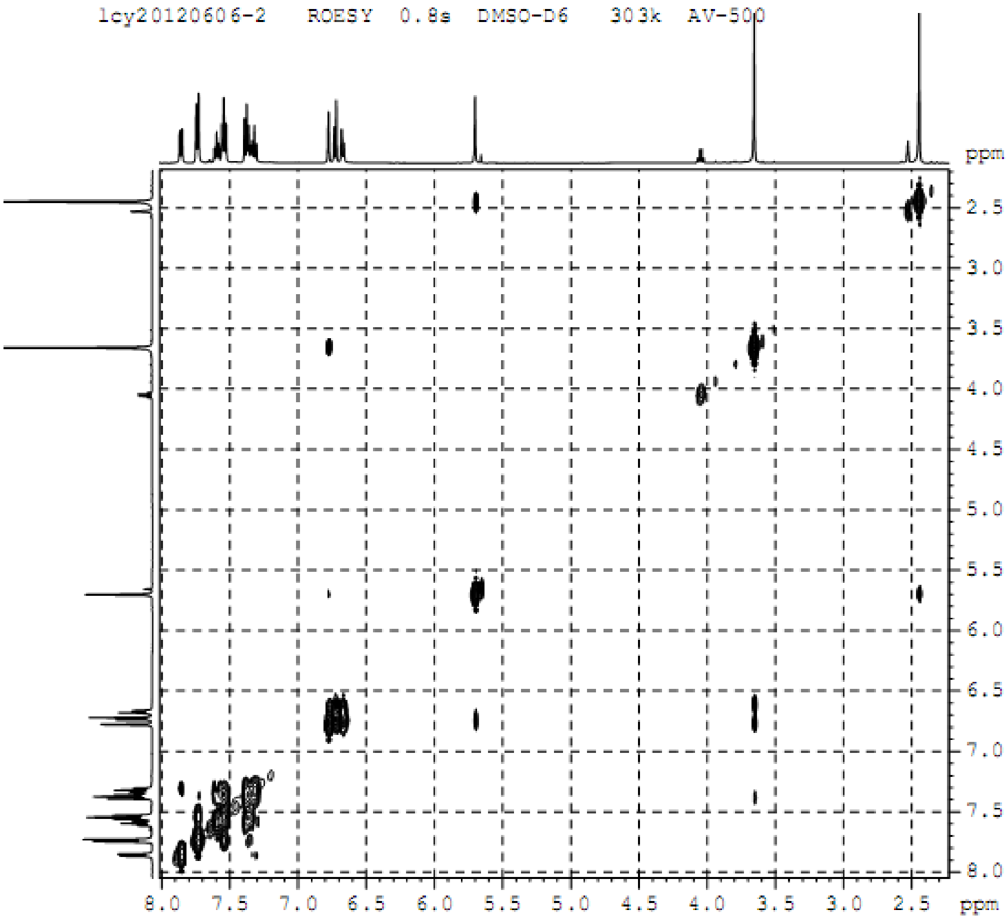
lcy20120606-2 ROESY 0.8s DMSO-D6 303k AV-500
ppm
2.5
3.0
3.5
4.0
4.5
5.0
5.5
6.0
6.5
7.0
7.5
8.0
8.0 7.5 7.0 6.5 6.0 5.5 5.0 4.5 4.0 3.5 3.0 2.5 ppm

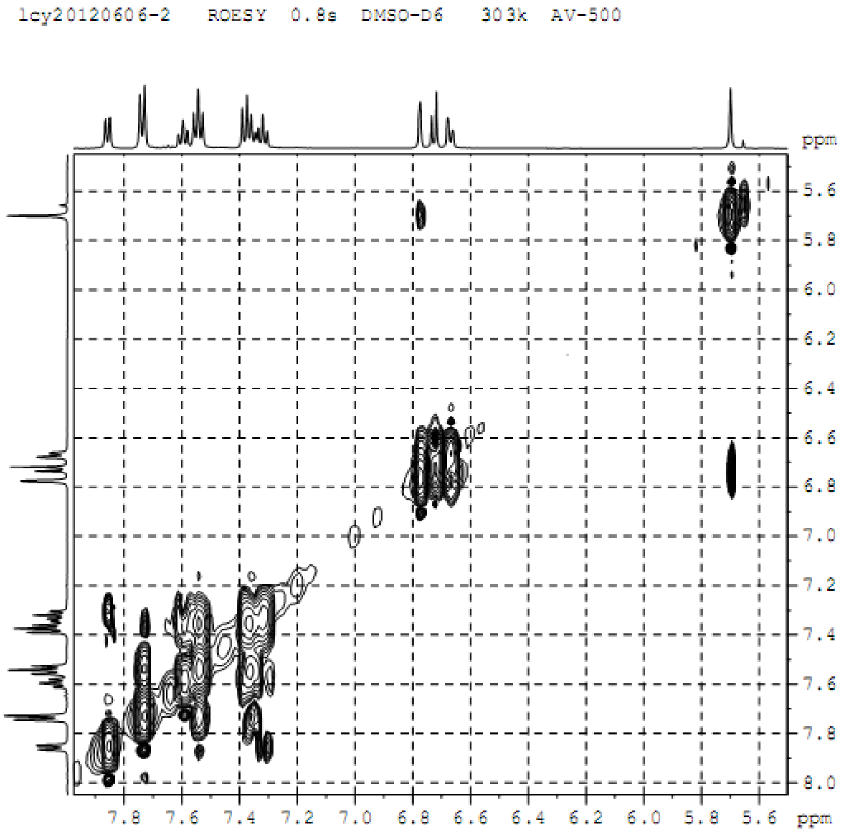
lcy20120606-2 ROESY 0.8s DMSO-D6 303k AV-500
ppm
5.6
5.8
6.0
6.2
6.4
6.6
6.8
7.0
7.2
7.4
7.6
7.8
8.0
7.8 7.6 7.4 7.2 7.0 6.8 6.6 6.4 6.2 6.0 5.8 5.6 ppm

Figure S5. DEPT spectrum of compound **4a**.

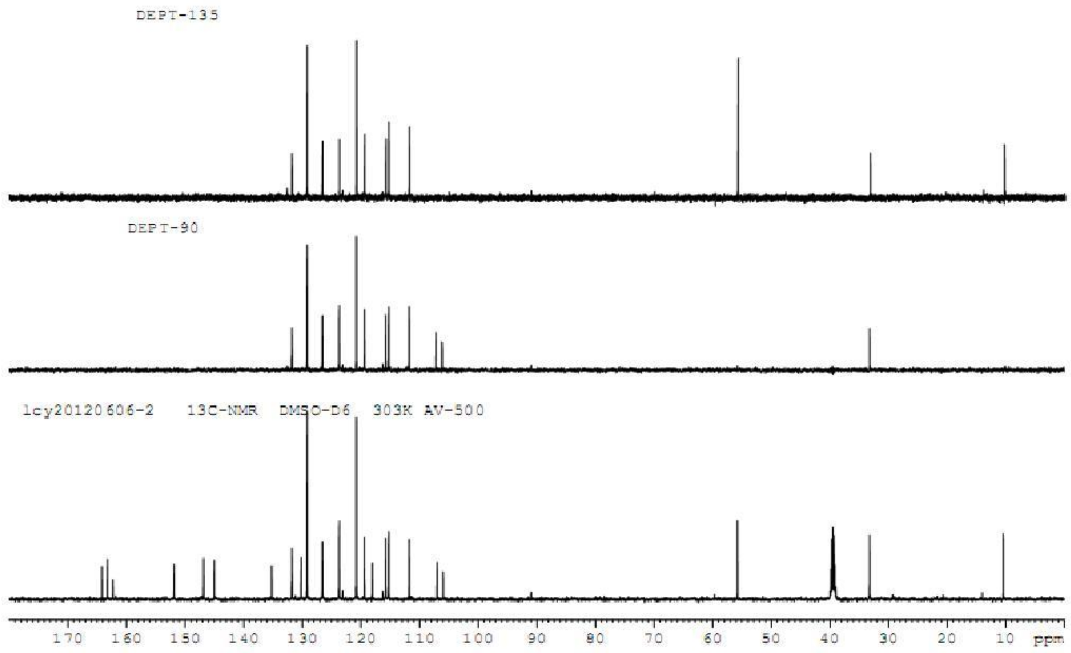

Figure S6. H-H COSY spectrum of compound **4a**.

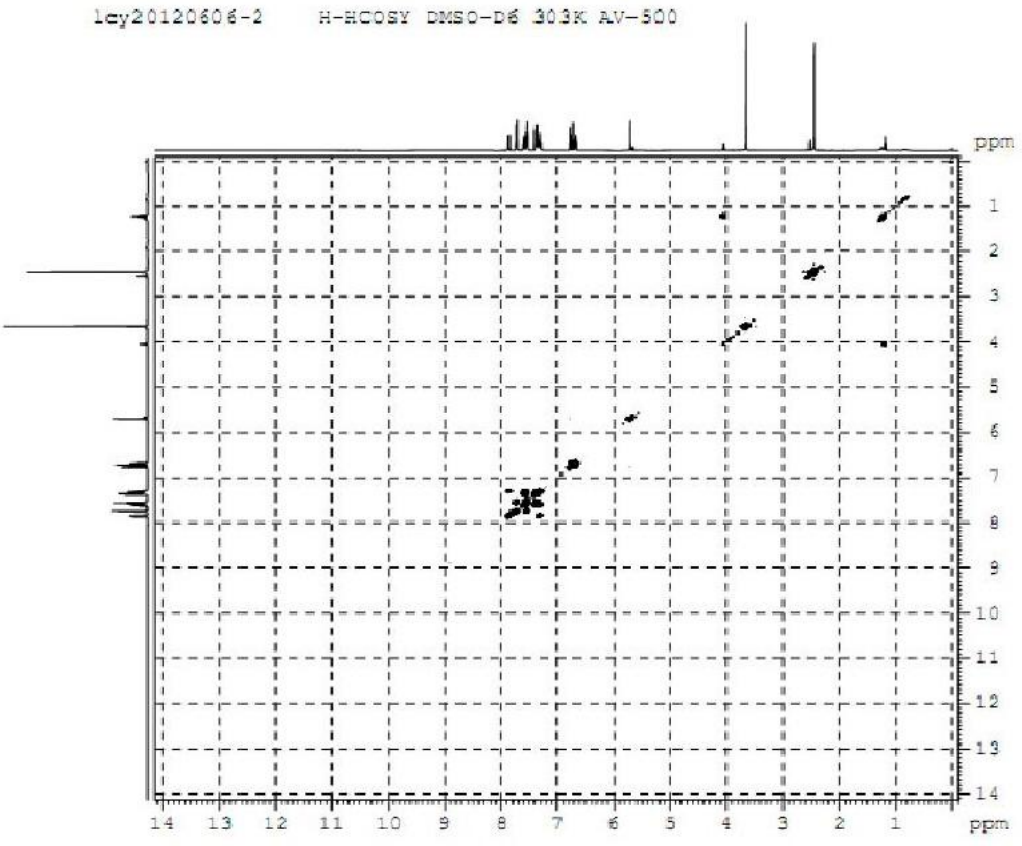

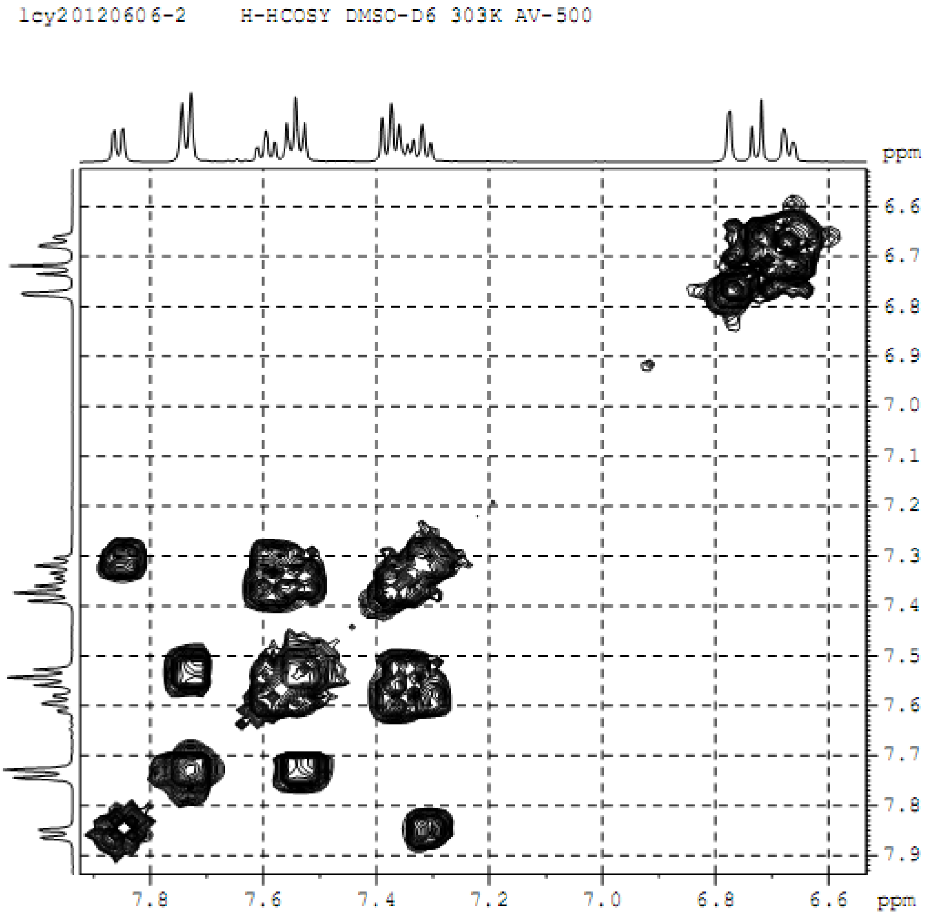

Figure S7. HMBC spectrum of compound **4a**.

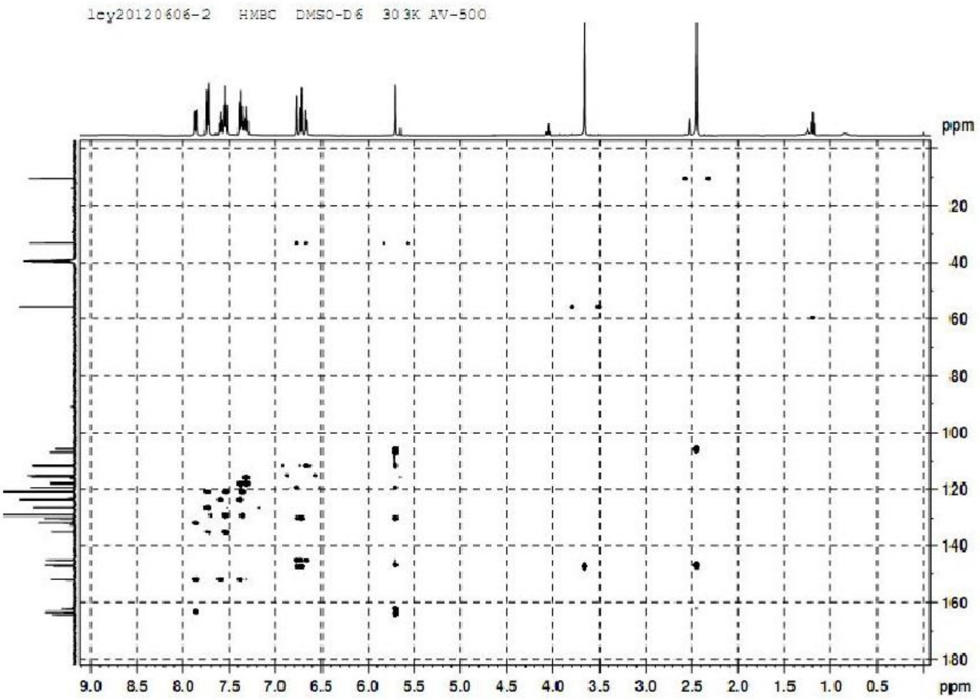

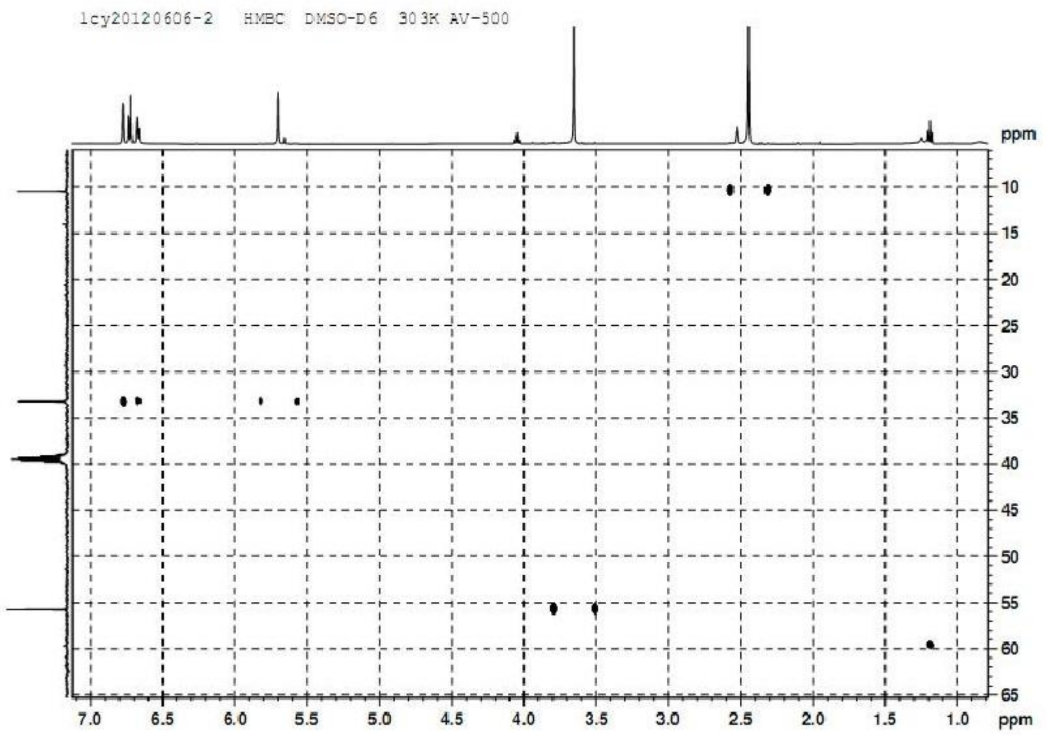
1cy20120606-2 HMBC DMSO-D6 303K AV-500
ppm
10
15
20
25
30
35
40
45
50
55
60
65
7.0
6.5
6.0
5.5
5.0
4.5
4.0
3.5
3.0
2.5
2.0
1.5
1.0
ppm

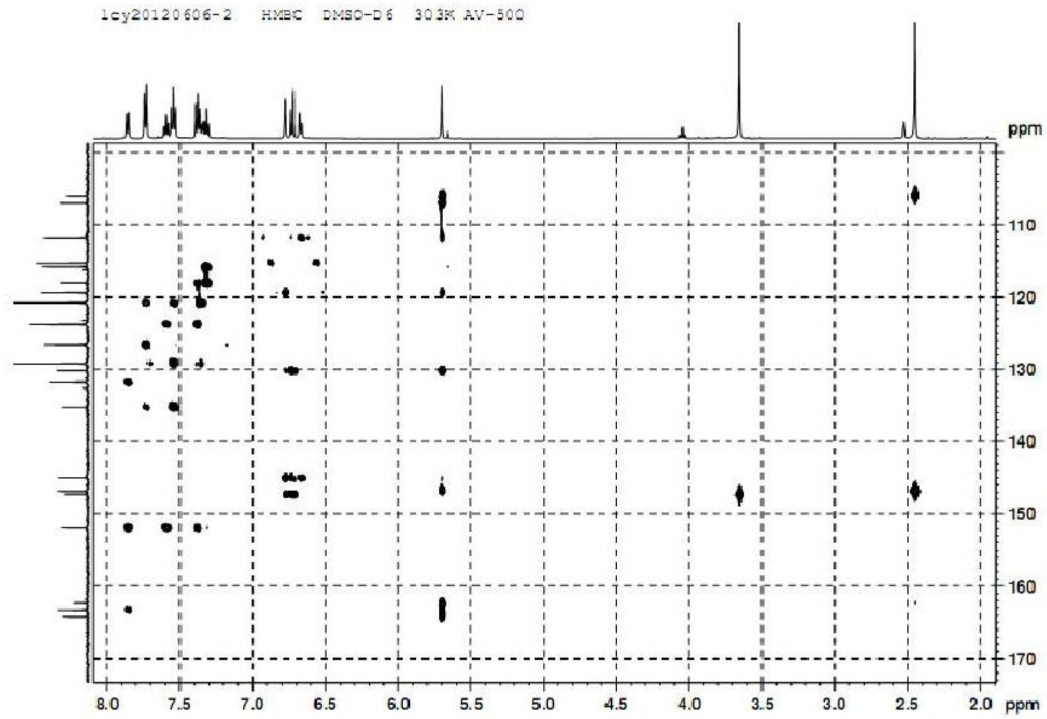
1cy20120606-2 HMBC DMSO-D6 303K AV-500
ppm
110
120
130
140
150
160
170
8.0
7.5
7.0
6.5
6.0
5.5
5.0
4.5
4.0
3.5
3.0
2.5
2.0
ppm

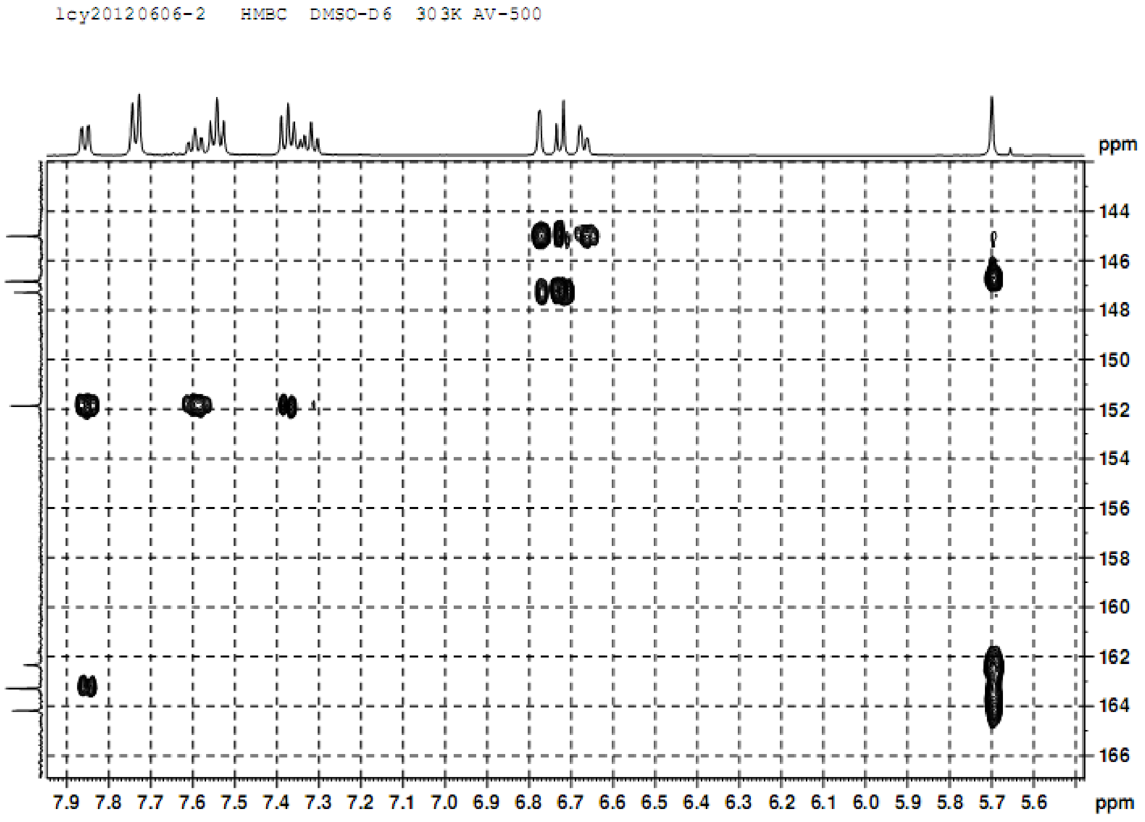
lcy20120606-2 HMBC DMSO-D6 303K AV-500
ppm
144
146
148
150
152
154
156
158
160
162
164
166
7.9 7.8 7.7 7.6 7.5 7.4 7.3 7.2 7.1 7.0 6.9 6.8 6.7 6.6 6.5 6.4 6.3 6.2 6.1 6.0 5.9 5.8 5.7 5.6
ppm

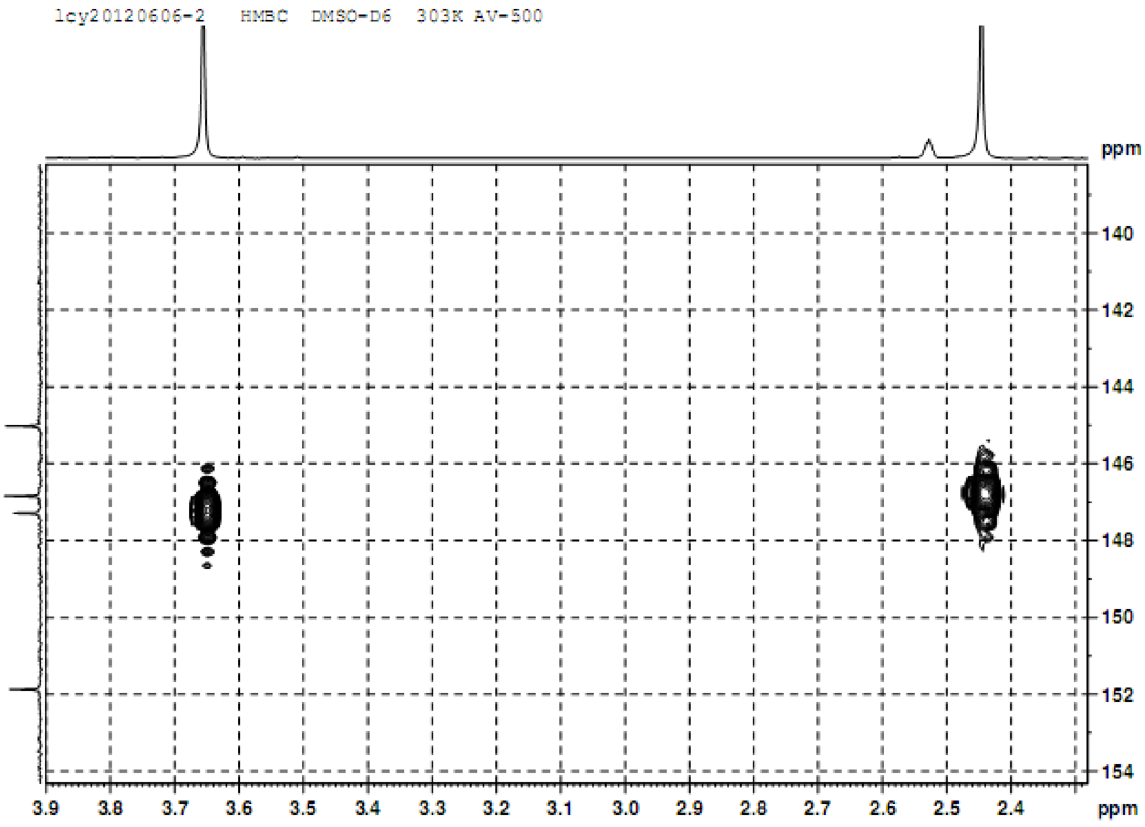
lcy20120606-2 HMBC DMSO-D6 303K AV-500
ppm
140
142
144
146
148
150
152
154
3.9 3.8 3.7 3.6 3.5 3.4 3.3 3.2 3.1 3.0 2.9 2.8 2.7 2.6 2.5 2.4
ppm

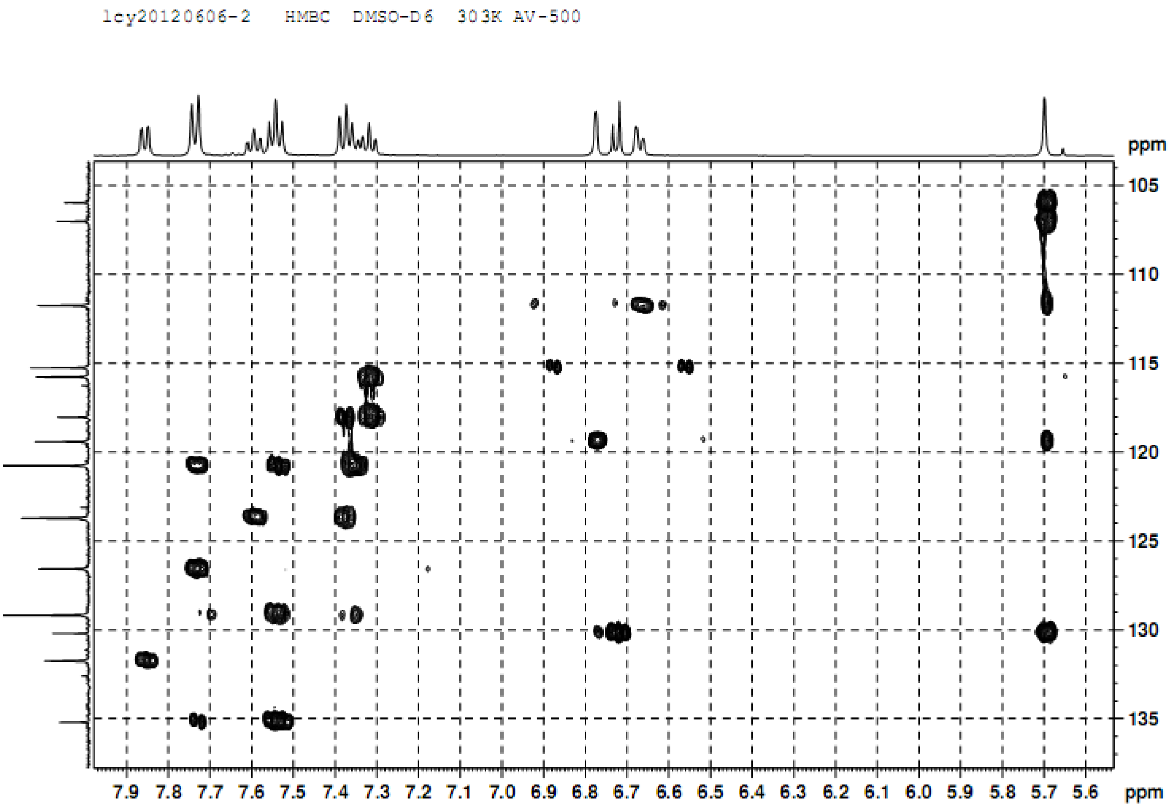
lcy20120606-2 HMBC DMSO-D6 303K AV-500
ppm
105
110
115
120
125
130
135
7.9 7.8 7.7 7.6 7.5 7.4 7.3 7.2 7.1 7.0 6.9 6.8 6.7 6.6 6.5 6.4 6.3 6.2 6.1 6.0 5.9 5.8 5.7 5.6
ppm
